# Author Correction: A phenotypic and genomics approach in a multi-ethnic cohort to subtype systemic lupus erythematosus

**DOI:** 10.1038/s41467-020-15097-z

**Published:** 2020-02-27

**Authors:** Cristina M. Lanata, Ishan Paranjpe, Joanne Nititham, Kimberly E. Taylor, Milena Gianfrancesco, Manish Paranjpe, Shan Andrews, Sharon A. Chung, Brooke Rhead, Lisa F. Barcellos, Laura Trupin, Patricia Katz, Maria Dall’Era, Jinoos Yazdany, Marina Sirota, Lindsey A. Criswell

**Affiliations:** 1 Russell/Engleman Rheumatology Research Center, Department of Medicine, University of California San Francisco, San Francisco, CA USA; 2 Bakar Computational Health Sciences Institute, University of California, San Francisco, CA USA; 3 Icahn School of Medicine at Mount Sinai, New York, NY USA; 4 University of California, Berkeley, CA USA; 5 Institute for Human Genetics, University of California, San Francisco, San Francisco, CA USA

**Keywords:** Computational biology and bioinformatics, Genetics, Epigenetics, Systemic lupus erythematosus

Correction to: *Nature Communications* 10.1038/s41467-019-11845-y, published online 29 August 2019.

In the original version of this manuscript, in the discussion section in the seventh paragraph, the gene symbol for *PARP14* was incorrectly given as *PAR14* and incorrect citations of the literature were given. The incorrect version read ‘We would like to highlight variants in *HLA-F*, *PAR14* and *GAB2* controlled methylation sites in *USP35*. *HLA-F* is part of the nonclassical *HLA-Ib* genes, which are mono- or oligomorphic^46^. Surface expression of *HLA-F* has been demonstrated on activated T, B and NK cells, and serum IgG autoantibodies against *HLA-F* have been detected in SLE patients and correlated with disease activity^63–65^. *PARP14* encodes for poly(ADP-ribose) polymerase (PARP) protein family 14 and is involved in cellular maintenance and cell fate decisions, such as cell-cycle progression, metabolic pathways and ribosome biogenesis^66^. Its role in SLE and autoimmune disease has not been defined but it has been shown to regulate glycolysis via IL-4 in B lymphocytes^67^ and to promote survival of cancer cells^67–69^.’

The correct version replaces these sentences with ‘We would like to highlight variants in *HLA-F*, *PARP14* and *GAB2* controlled methylation sites in *USP35*. *HLA-F* is part of the nonclassical *HLA-Ib* genes, which are mono- or oligomorphic^46^. Surface expression of *HLA-F* has been demonstrated on activated T, B and NK cells, and serum IgG autoantibodies against *HLA-F* have been detected in SLE patients and correlated with disease activity^63–65^. *PARP14* encodes for poly(ADP-ribose) polymerase (PARP) protein family 14 and assists in post-translational ribosylation modification of target proteins. Its role in SLE and autoimmune disease has not been defined but it has been shown to regulate glycolysis via IL-4 in B lymphocytes^66^, promote survival of cancer cells^67^, and regulate macrophage activation^68^.’

Further, the original refs. ^66–69^ were replaced with the following corrected refs. ^66–68^ and all following references were renumbered.

All of these errors have now been corrected in the HTML and PDF versions of the article.
