# Supplementary material for: A phenotypic and genomics approach in a multi-ethnic cohort to subtype systemic lupus erythematosus
Source: Nat Commun. 2019 Aug 29;10:3902. doi: 10.1038/s41467-019-11845-y (PMC6715644; doi:10.1038/s41467-019-11845-y)
Supplement: Supplementary file 1 — Supplementary Information [file 41467_2019_11845_MOESM1_ESM.docx]

**A phenotypic and genomics approach in a multi-ethnic cohort to subtype systemic lupus erythematosus**

Lanata et al.

**Supplementary Information**

**Supplementary Tables**

**Supplementary Table 1. Characteristics of the 333 patients in the UCSF CLUES cohort**

| **Characteristic** | **Value** |
| --- | --- |
| Number of Female Subjects (%) | 298 (88.9) |
| Age age blood draw, mean years (SD) | 45 (13.9) |
| Disease Duration, mean years (SD) | 16.3 (10.5) |
| SLEDAI at blood draw (SD) | 3 (3.1) |
| Self-Reported Race |  |
| White | 96 (28.8) |
| Hispanic | 76 (22.8) |
| African-American | 36 (10.8) |
| Asian | 118 (35.4) |
| Other | 7 (2.1) |
| **ACR SLE Classification Criteria, n (%)** |  |
| ACR Malar Rash | 150 (45) |
| ACR Discoid Rash | 39 (11.7) |
| ACR Oral Ulcers | 150 (45) |
| ACR Photosensitivity | 134 (40.2) |
| ACR Arthritis | 265 (79.6) |
| ACR Serositis | 137 (41.1) |
| ACR Pleuritis | 105 (31.6) |
| ACR Pericarditis | 62 (18.7) |
| ACR Neurologic | 34 (10.2) |
| ACR Seizure | 22 (6.6) |
| ACR Psychosis | 13 (3.9) |
| ACR Immunologic | 269 (80.8) |
| ACR anti-dsDNA | 225 (67.6) |
| ACR anti-Smith | 98 (29.4) |
| ACR ANA | 319 (95.8) |
| ACR Hematologic | 156 (46.8) |
| ACR Hemolytic Anemia | 26 (7.8) |
| ACR Leukopenia | 73 (21.9) |
| ACR Lymphopenia | 106 (31.8) |
| ACR Thrombocytopenia | 56 (16.8) |

|  | **Cluster** | | |  |
| --- | --- | --- | --- | --- |
|  | M (N=101) | S1 (N=154) | S2 (N=78) | P Value |
| **Current Medications (% of patients in cluster)** |  |  |  |  |
| mycophenolate | 24.24% | 45.27% | 41.03% | 2.48E-03 |
| gabapentin | 13.13% | 3.38% | 5.13% | 1.29E-02 |
| folic Acid | 15.15% | 6.08% | 15.38% | 2.55E-02 |
| albuterol | 10.10% | 2.70% | 3.85% | 4.59E-02 |
| pantoprazole | 10.10% | 2.70% | 5.13% | 4.77E-02 |
| prednisone | 44.44% | 53.38% | 62.82% | 5.07E-02 |
| multi-vitamin | 13.13% | 15.54% | 5.13% | 6.54E-02 |
| calcium with vitamin D | 15.15% | 7.43% | 15.38% | 7.97E-02 |
| benazepril | 2.02% | 8.11% | 3.85% | 9.36E-02 |
| azathioprine | 8.08% | 13.51% | 19.23% | 9.39E-02 |
| **Current Blood Labs** |  |  |  |  |
| C3 (SD) | 106.56 (31.16) | 94.19 (29.63) | 90.92 (32.2) | 1.47E-03 |
| C Reactive Protein (SD) | 3.74 (8.51) | 2.62 (6.37) | 2.66 (4.89) | 4.28E-01 |
| Serum Creatinine (SD) | 0.88 (0.71) | 1.2 (1.82) | 1.26 (1.59) | 1.71E-01 |
| RNP (% of patients positive) | 24.44% | 34.75% | 58.11% | 3.66E-05 |
| anti-SSA (% of patients positive) | 23.40% | 49.32% | 45.45% | 2.11E-04 |
| anti-SSB (% of patients positive) | 5.26% | 13.51% | 12.99% | 1.06E-01 |
| ANA (% of patients positive) | 63.54% | 69.39% | 81.58% | 3.32E-02 |
| anti-Sm (% of patients positive) | 11.58% | 23.29% | 44.16% | 4.73E-06 |
| anti-dsDNA (% of patients positive) | 26.53% | 47.62% | 71.83% | 3.95E-08 |
| **Current CBC** |  |  |  |  |
| WhiteBloodCell (SD) | 6.23 (2.16) | 5.95 (2.28) | 5.15 (1.92) | 3.31E-03 |
| AbsNeutro (SD) | 3094.38 (2304.36) | 3206.92 (2101.65) | 3013.42 (2127.23) | 8.05E-01 |
| AbsLympho (SD) | 1162.79 (861.05) | 1052.49 (687.94) | 864.79 (654.14) | 2.84E-02 |
| AbsMono (SD) | 339.72 (281.2) | 340.14 (227.94) | 292.97 (227.89) | 3.39E-01 |
| AbsEosino (SD) | 86.2 (95.92) | 79.45 (87.18) | 53.97 (58.64) | 3.09E-02 |
| AbsBaso (SD) | 24.55 (21.07) | 21.53 (19.19) | 19.18 (23.9) | 2.30E-01 |

**Supplementary Table 2** **Summary of medications and laboratory values at the time of blood sampling for patients in three clinical clusters**. P-values were computed using ANOVA for continuous variables and Fisher exact test for categorical variables.

**Supplementary Table 3** **Summary of socioeconomic and exposure variables for patients in three clinical clusters**. P-values were computed using ANOVA for continuous variables (education, income) and Fisher’s exact test for categorical variables.

|  | **Cluster** | | |  |
| --- | --- | --- | --- | --- |
|  | M (N=101) | S1 (N=154) | S2 (N=78) | P Value |
| Education (SD) | 4.28 (1.49) | 3.81 (1.64) | 3.81 (1.67) | 0.05066 |
| Income (SD) | 4.03 (1.81) | 3.64 (1.93) | 3.43 (2) | 0.11631 |
| Exposure to Bug Spray (% of patients positive) | 26.00% | 18.00% | 23.40% | 0.27502 |
| Exposure to Agriculture | 36.40% | 19.50% | 32.10% | 0.00723 |
| Exposure to Kerosene (% of patients positive) | 6.00% | 7.90% | 15.60% | 0.08719 |
| Smoke Exposure in Childhood (% of patients positive) | 70.00% | 57.50% | 41.00% | 0.00053 |
| Smoke Exposure at Home (% of patients positive) | 16.70% | 9.80% | 15.10% | 0.25378 |

| Education and income variables were coded using a categorical scale as below: | | | | |
| --- | --- | --- | --- | --- |
|  |  |  |  |  |
| **Variable Keys** | | | | |
| **Education:** |  |  | **Income:** |  |
| Less than high school (1) | |  | Less than $20,000 (1) | |
| High school graduate (2) | |  | $20,000 - < $40,000 (2) | |
| Some college/no degree (3) | |  | $40,000 - < $60,000 (3) | |
| Associate degree/trade or vocational school (4) | |  | $60,000 - < $80,000 (4) | |
| College graduate (Bachelor's degree) (5) | |  | $80,000 - < $400,000 (5) | |
| Masters/PhD/professional degree (6) | |  | $400,000 or more (6) | |

**Supplementary Table 4 Clinical Characteristics of Validation Dataset Clusters.** Cluster labels were assigned using predictions from random forest model trained on original CLUES data clusters. ACR criteria were compared across clusters using chi-squared test. Lupus severity index was compared using ANOVA test.

|  | **Cluster** | | |  |  |
| --- | --- | --- | --- | --- | --- |
|  | M (N=164) | S1 (N=76) | S2 (N=86) | P Value | FDR |
| **ACR Criteria** |  |  |  |  |  |
| ACR Leukopenia | 11.0% | 13.2% | 73.3% | 1.14E-26 | 2.05E-25 |
| ACR anti-dsDNA | 20.1% | 69.7% | 83.7% | 1.83E-24 | 1.65E-23 |
| ACR Lymphoenia | 47.6% | 26.3% | 94.2% | 1.37E-18 | 8.25E-18 |
| ACR Photosensitivity | 98.2% | 52.6% | 72.1% | 6.33E-17 | 2.85E-16 |
| ACR Renal | 5.5% | 40.8% | 46.5% | 6.28E-15 | 2.26E-14 |
| ACR anti-Smith | 3.0% | 7.9% | 30.2% | 5.59E-10 | 1.68E-09 |
| ACR Malar Rash | 57.9% | 17.1% | 52.3% | 1.44E-08 | 3.70E-08 |
| ACR Oral Ulcers | 42.1% | 9.2% | 26.7% | 1.21E-06 | 2.72E-06 |
| ACR Arthritis | 81.7% | 56.6% | 81.4% | 4.83E-05 | 9.66E-05 |
| ACR Thrombocytopenia | 6.7% | 14.5% | 25.6% | 1.76E-04 | 3.16E-04 |
| ACR APLA | 28.7% | 53.9% | 47.7% | 1.94E-04 | 3.17E-04 |
| ACR Hemolytic Anemia | 2.4% | 5.3% | 12.8% | 3.94E-03 | 5.91E-03 |
| ACR ANA | 93.3% | 100.0% | 93.0% | 6.52E-02 | 9.03E-02 |
| ACR Seizure | 5.5% | 6.6% | 12.8% | 1.10E-01 | 1.41E-01 |
| ACR Pleuritis | 26.2% | 18.4% | 26.7% | 3.66E-01 | 4.39E-01 |
| ACR Discoid Rash | 3.0% | 1.3% | 4.7% | 4.70E-01 | 5.29E-01 |
| ACR Pericarditis | 10.4% | 9.2% | 14.0% | 5.83E-01 | 6.18E-01 |
| ACR Psychosis | 1.8% | 1.3% | 2.3% | 8.92E-01 | 8.92E-01 |
| **Lupus Severity Index (SD)** | 5.4 (0.95) | 6.8 (1.4) | 6.8 (1.6) | 1.36E-18 | 1.36E-18 |

**Supplementary Table 5 Methylation sites with evidence of mediation from causal inference testing.**

|  |  |  |  |  | meQTL Model | | | CIT Model | |
| --- | --- | --- | --- | --- | --- | --- | --- | --- | --- |
| SNP | CpG | CPG Gene | SNP gene | rs_id | P value | FDR | beta | P value | FDR |
| 11.7800717 | cg07259759 | USP35 | GAB2 | rs7104222 | 5.46E-19 | 2.99E-14 | 0.17283424 | 0.00201999 | 0.0049887 |
| 11.7812458 | cg07259759 | USP35 | GAB2 | rs10899498 | 1.22E-16 | 2.00E-12 | 0.15534721 | 0.00322565 | 0.0061456 |
| 11.7804852 | cg07259759 | USP35 | GAB2 | rs2063730 | 1.10E-15 | 1.51E-11 | 0.15589434 | 0.00038023 | 0.0049887 |
| 11.7813523 | cg07259759 | USP35 | LOC101928865 | rs11237490 | 4.49E-13 | 2.38E-09 | 0.14481098 | 0.0020943 | 0.0049887 |
| 6.29707661 | cg23892836 | HLA-F | HLA-F |  | 2.74E-11 | 9.36E-08 | 0.22412686 | 0.00173717 | 0.0049887 |
| 11.7792804 | cg07259759 | USP35 | GAB2 |  | 5.13E-10 | 1.11E-06 | 0.11447828 | 0.00375862 | 0.00726008 |
| 11.7811705 | cg07259759 | USP35 | GAB2 | rs6592775 | 3.38E-08 | 3.77E-05 | 0.10391034 | 0.00222163 | 0.0049887 |
| 6.29707661 | cg11617938 | HLA-F | HLA-F |  | 8.02E-08 | 7.39E-05 | 0.18183347 | 0.01502458 | 0.01645038 |
| 6.29707661 | cg15331332 | HLA-F | HLA-F |  | 2.79E-07 | 0.00022107 | 0.21130355 | 0.0022019 | 0.0049887 |
| 11.7797941 | cg07259759 | USP35 | GAB2 | rs7927923 | 2.91E-07 | 0.00022399 | -0.0960436 | 0.00177115 | 0.0049887 |
| 6.29707661 | cg24351901 | HLA-F | HLA-F |  | 6.03E-07 | 0.00039411 | 0.15576723 | 0.00173717 | 0.0049887 |
| 11.7824948 | cg07259759 | USP35 | LOC101928896 | rs12419623 | 1.72E-05 | 0.00677514 | -0.086491 | 0.00836239 | 0.00767528 |
| 22.5084864 | cg11224765 | ODF3B | PPP6R2 | rs112430043 | 4.64E-05 | 0.01514605 | 0.40985225 | 0.00228763 | 0.0049887 |
| 3.12241875 | cg00272009 | PARP14 | PARP14 | rs11719086 | 6.29E-05 | 0.0189825 | 0.24220456 | 0.00752833 | 0.00767528 |
| 20.6222934 | cg00480970 | HELZ2 | GMEB2 |  | 8.82E-05 | 0.02503083 | 0.13072551 | 0.00210834 | 0.0049887 |
| 3.46042073 | cg24954967 | NA | FYCO1 |  | 9.01E-05 | 0.02523446 | 0.18283376 | 0.00072094 | 0.0049887 |
| 11.5378507 | cg12461141 | TRIM22 | OR51B5 | rs12421528 | 0.00010227 | 0.02764241 | 0.2500682 | 0.0006956 | 0.0049887 |
| 6.29571609 | cg24351901 | HLA-F | GABBR1 | rs10946999 | 0.00013337 | 0.03418973 | -0.1508784 | 0.02105528 | 0.01645038 |
| 17.9422425 | cg05726450 | PIK3R6 | STX8 |  | 0.00015114 | 0.03745947 | -0.0845213 | 0.05075674 | 0.04434778 |
| 12.1134189 | cg04708790 | OAS1 | OAS2 |  | 0.0001585 | 0.0387288 | -0.1169917 | 0.00947887 | 0.00767528 |
| 12.1131929 | cg04708790 | OAS1 | RPH3A |  | 0.00018142 | 0.04218172 | -0.0963112 | 0.00864478 | 0.00767528 |
| 12.1135014 | cg25800166 | OAS3 | NA | NA | 0.00018606 | 0.04275485 | 0.18167606 | 0.00304492 | 0.0061456 |
| 12.1131376 | cg04708790 | OAS1 | RPH3A |  | 0.00019841 | 0.04496177 | -0.1410518 | 0.00089759 | 0.0049887 |
| 8.14457685 | cg21995613 | NA | ZC3H3 |  | 0.00020337 | 0.04589732 | 0.32342071 | 0.00128402 | 0.0049887 |

**Supplementary Figure 1** **Quantile-Quantile Plot.** The -10log10 P values observed for association of CpGs with clinical cluster (blue dots) are plotted against the theoretical -10log10 P values expected under the null hypothesis (red line). The genomic inflation factor (l) was estimated to be 0.99.

Quantile-Quantile Plot and Genomic Inflation Factor


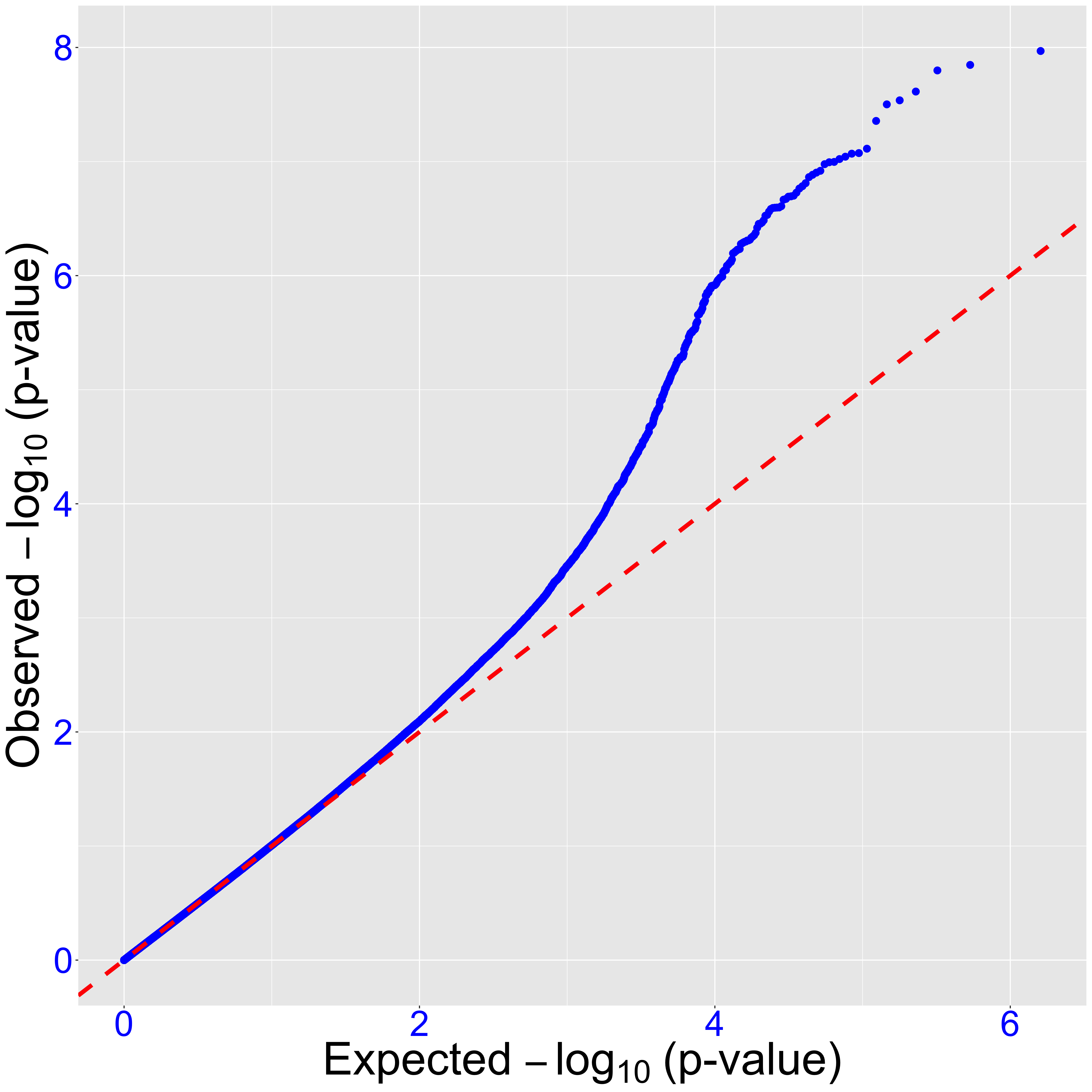


**Supplementary Figure 2 Cluster-Specific Methylation Signature. A.** CpG sites differentially methylated in cluster S2 vs M. **B.** CpG sites differentially methylated in cluster S2 vs S1. **C.** CpG sites differentially methylated in cluster S1 vs M. Red points indicates CpGs with FDR<0.1 and blue points indicates CpGs with ΔBeta >0.05 and FDR<0.1. Significance was assed using a nested F test as described in Methods.


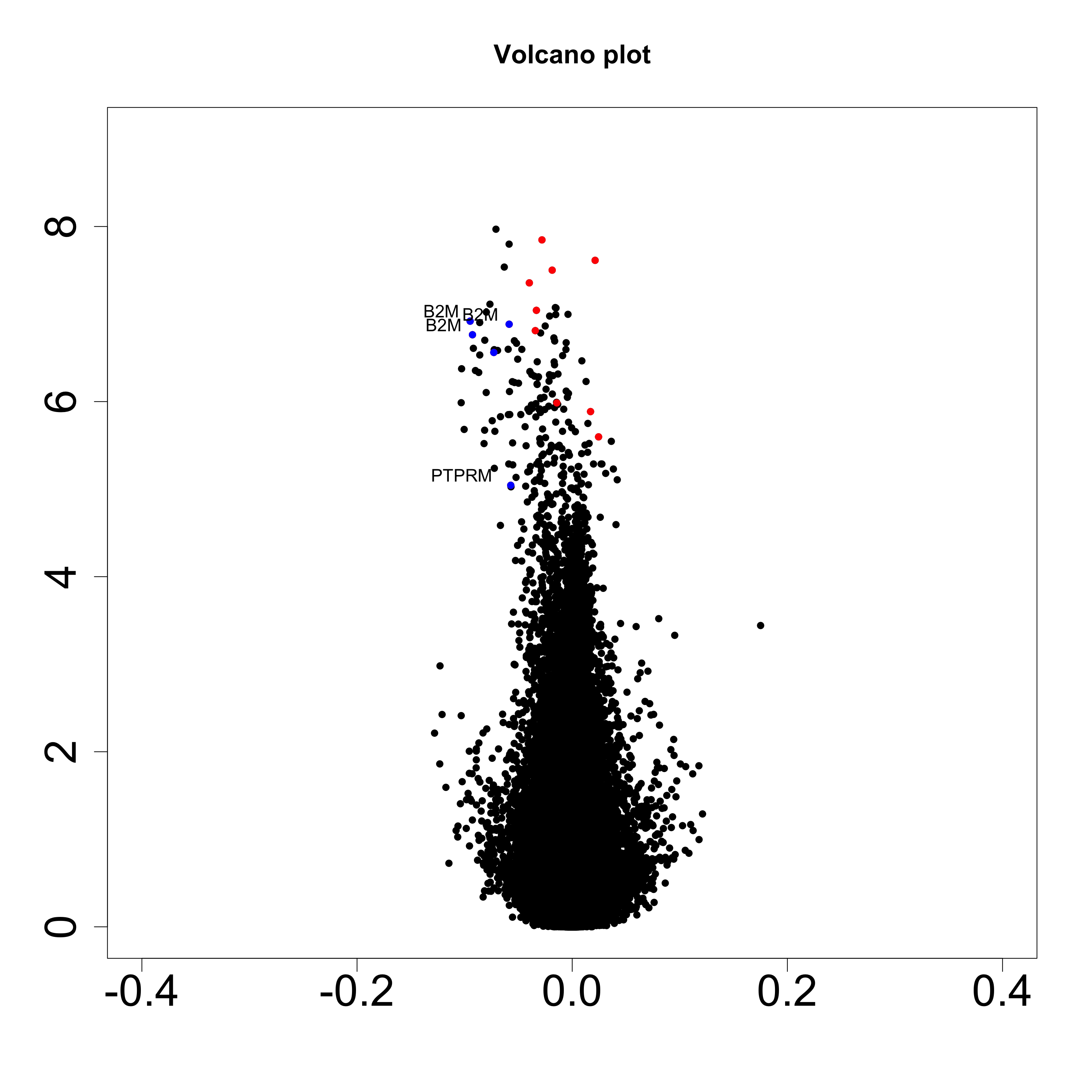

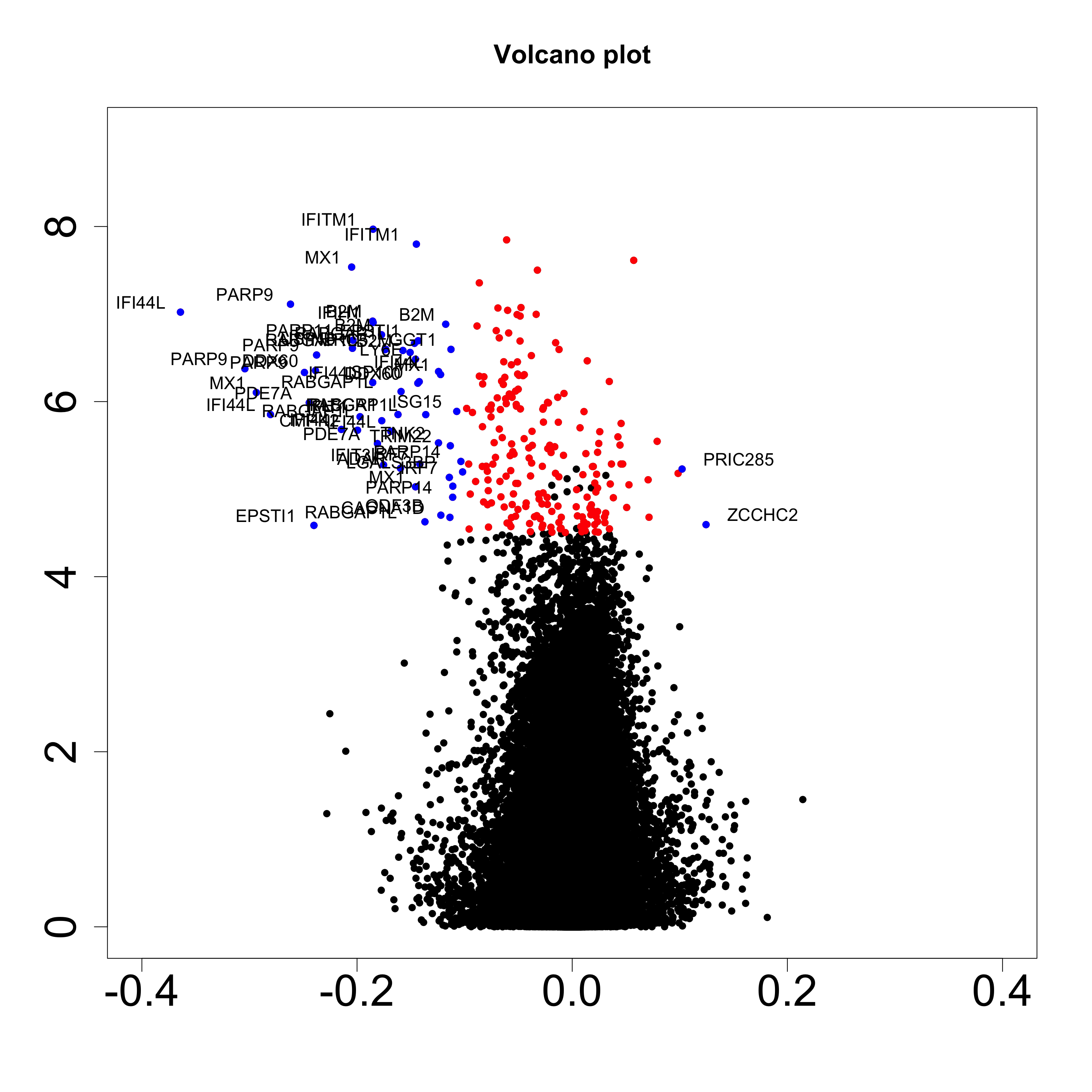

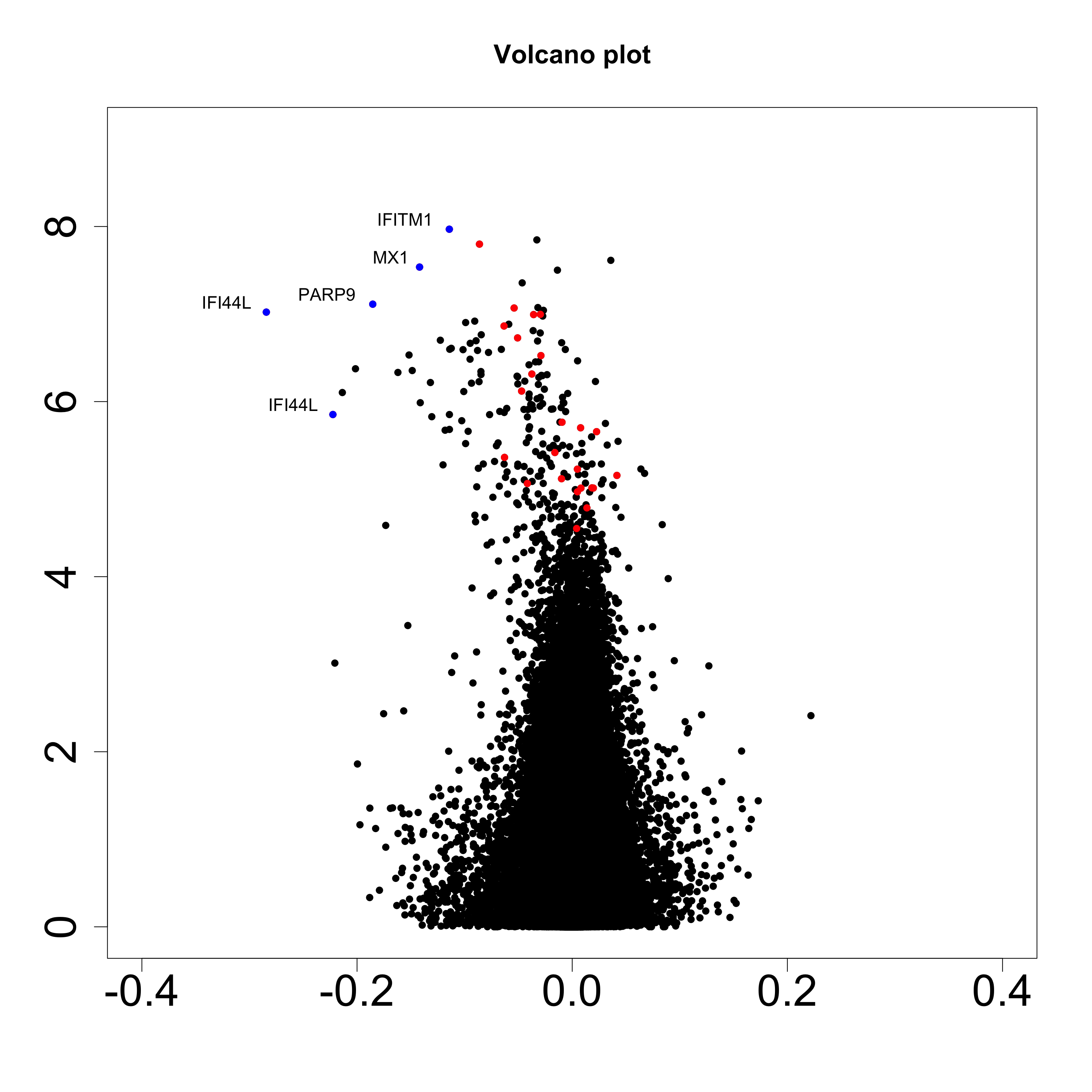


Cluster S1 vs M

**C**

Delta Beta

Cluster S2 vs S1

**B**

Delta Beta

-log10(P-value)

Delta Beta

-log10(P-value)

Cluster S2 vs M

**A**

-log10(P-value)

**Supplementary Figure 3 Random Forest Parameter Optimization.** A random forest model was fit to the 18 ACR clinical features to predict clinical cluster using the randomForest R package. The mtry variable was optimized by minimizing the OOB error over mtry ϵ [1,18].


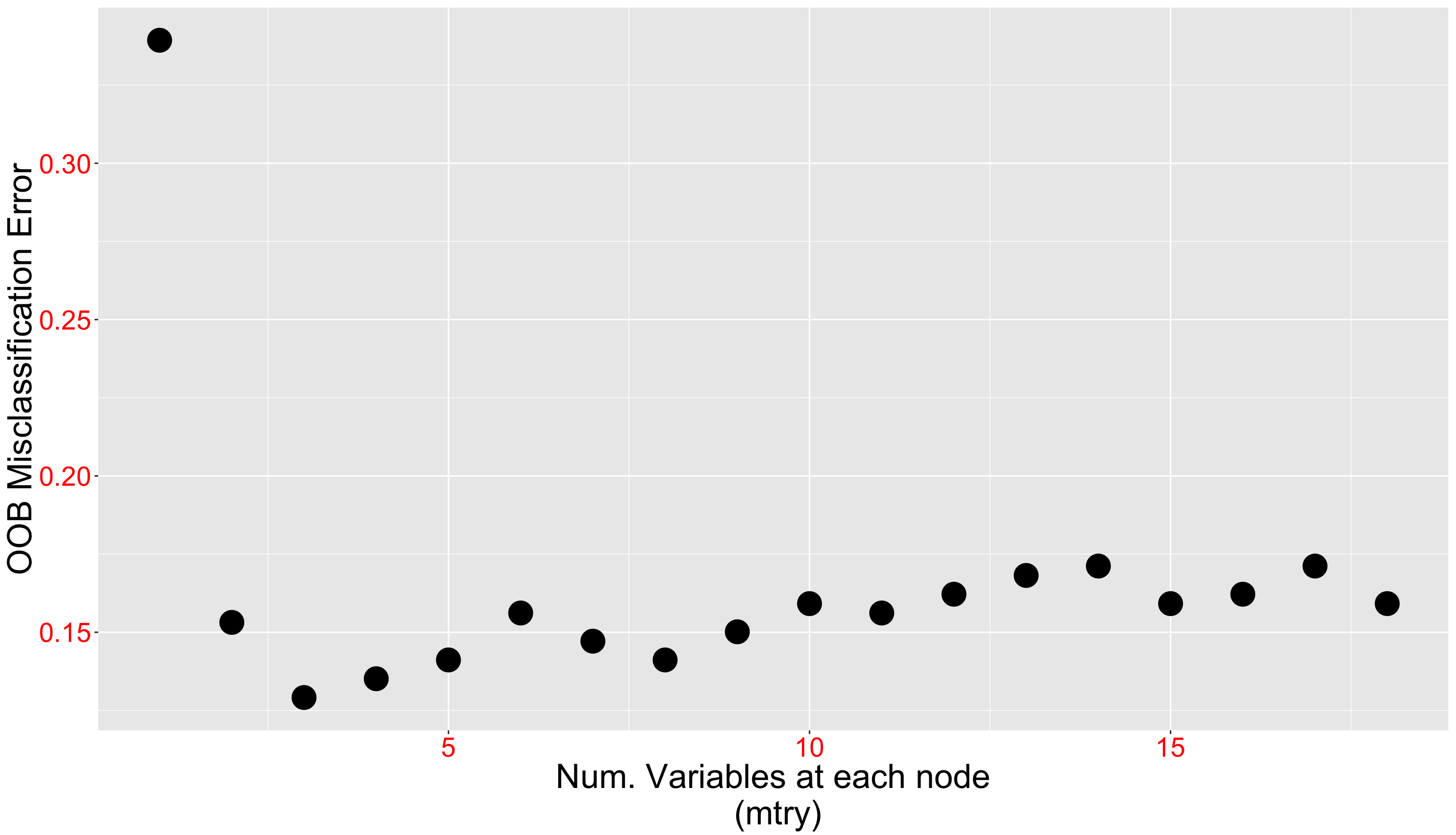


**Supplementary Figure 4 Enrichment/depletion of cluster associated CpGs in Epigenome Roadmap 15-state chromatin classification model in peripheral blood cells.** ([https://egg2.wustl.edu/roadmap/web_portal/chr_state_learning.html#core_15state)](https://egg2.wustl.edu/roadmap/web_portal/chr_state_learning.html). Testing was conducted by hypergeometric test significant (q<0.01) enrichment/depletion marked with an X. Colors indicate odds ratios of enrichment/depletion of chromatin state in cluster associated CpGs with respect to all CpGs on EPIC chip.


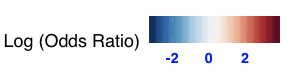


**Active States**

**Repressed States**

**Supplementary Figure 5** **Enrichment of cluster associated CpGs in Epigenome Roadmap histone ChIP peaks in peripheral blood cells.** Testing was conducted by hypergeometric test for enrichment of cluster associated CpG sites in ChIP-seq peaks with respect to all CpGs tested on Illumina EPIC chip.


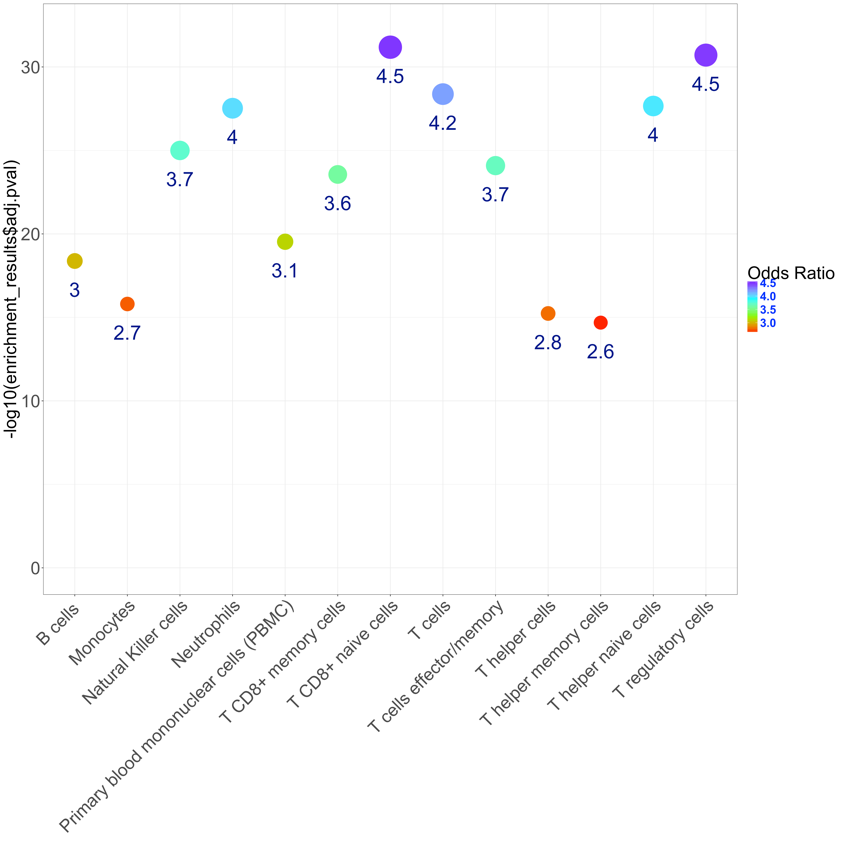

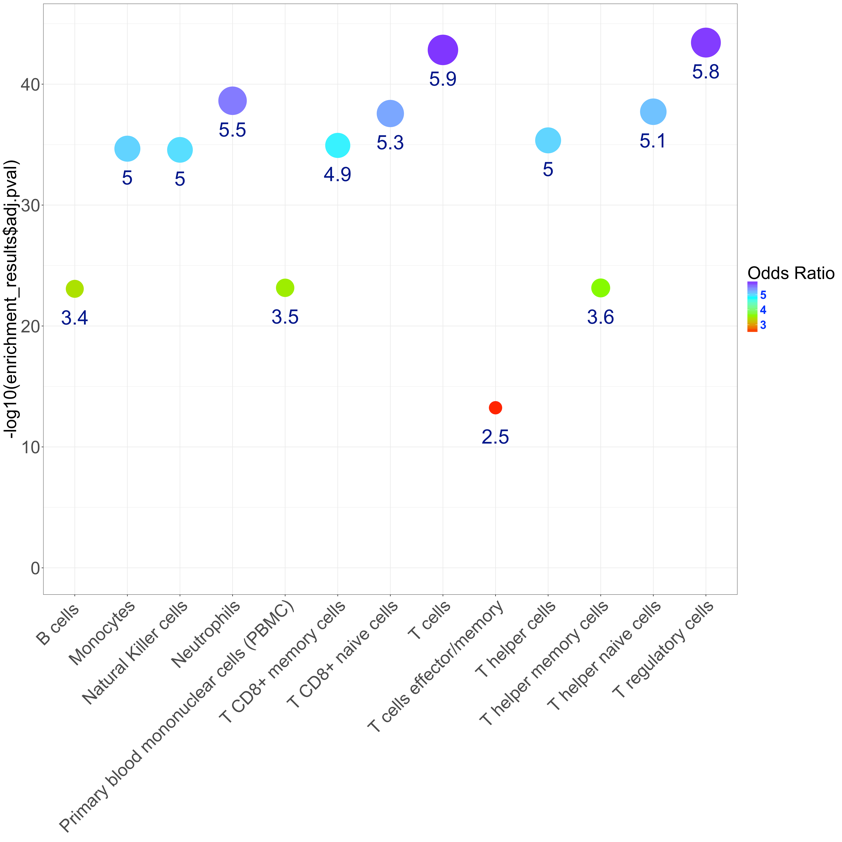

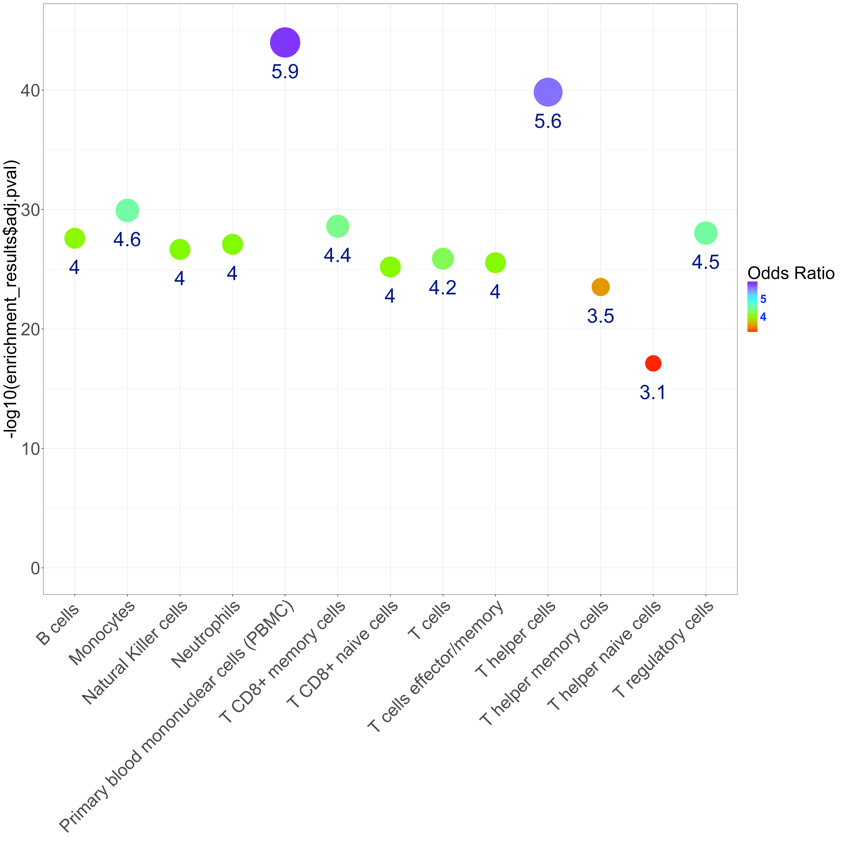


H3K4me3

H3K27ac

H3K4me1

**Supplementary Figure 6 Cluster-associated CpG meQTL Analysis. A.** Distribution of distance between SNPs and CpGs for significant meQTL associations (FDR<0.05, red) and non-significant meQTLs (blue). **B.** Distance between SNPs and CpGs is plotted against –log_10_(p-value) for significant meQTLs (FDR<0.05). Negative/positive distances denote that the SNP is located upstream/downstream of the CpG with respect to the positive strand of the reference genome.


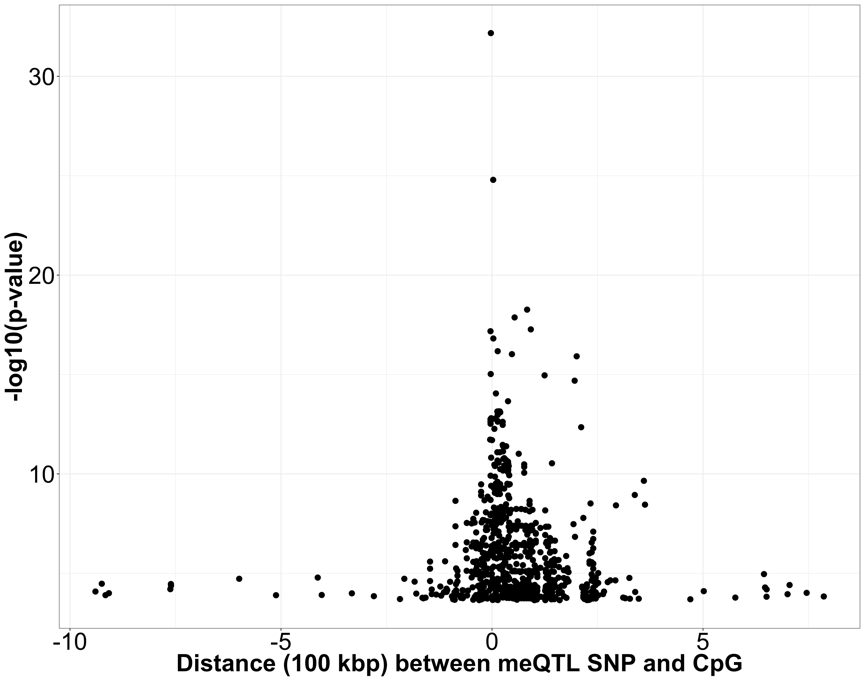

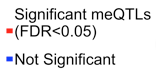

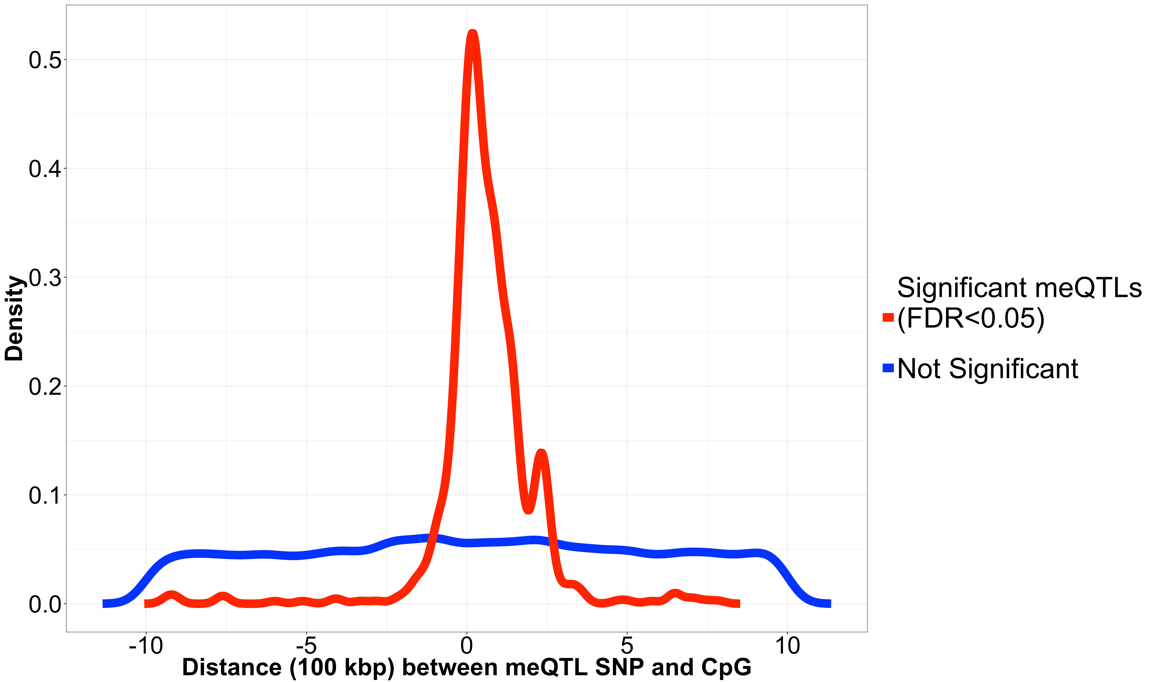


B

A

**Supplementary Figure 7 Unsupervised Clustering Methodology. A.** *MissMDA* R package was used to select the number of components to retain after performing multiple Correspondence analysis (MCA) on ACR data. Number of components that minimizes mean squared error of prediction was chosen. **B.** Stability of K-means cluster (k=3) evaluated using bootstrap resampling strategy (100 times) implemented in *fpc* R package. Boxplot displays distribution of 100 Jaccard similarity scores for clusters for each bootstrapped sample.


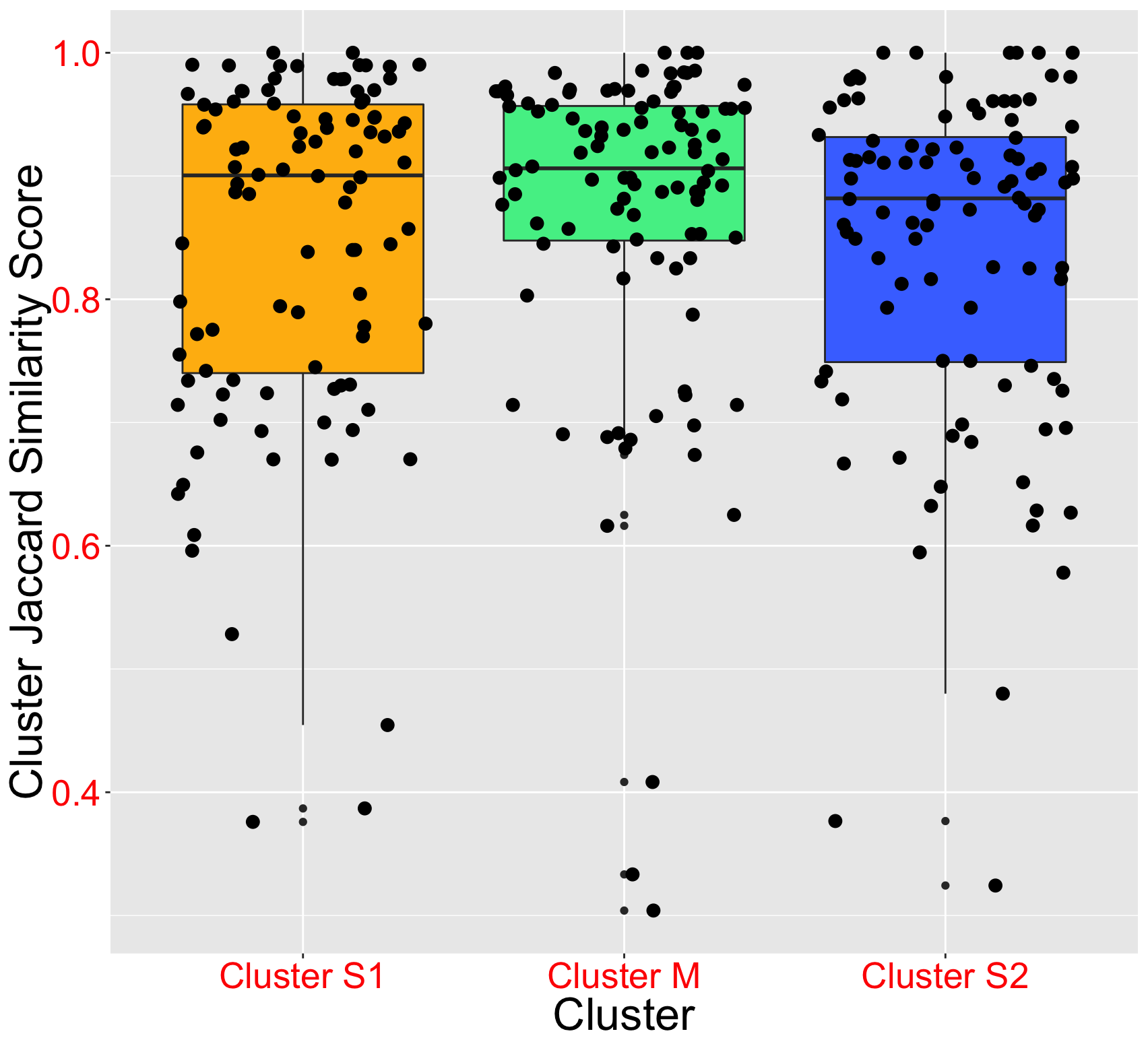


K-Means clustering Stability

MCA Components

**B**

**A**
