# Supplementary material for: A phenotypic and genomics approach in a multi-ethnic cohort to subtype systemic lupus erythematosus
Source: Nat Commun. 2019 Aug 29;10:3902. doi: 10.1038/s41467-019-11845-y (PMC6715644; doi:10.1038/s41467-019-11845-y)
Supplement: Supplementary file 5 — Description of Additional Supplementary Files [file 41467_2019_11845_MOESM5_ESM.docx]

**Title: Supplementary Data 1 List of all significant cluster-associated CpGs (FDR< 0.1).**
**Description:** Each CpG was mapped to a gene using the Illumina EPIC annotation and P-values were computed as described in Methods.

**Title: Supplementary Data 2** **List of all significant cluster-associated CpGs (FDR< 0.1) for pairwise cluster comparisons.**
**Description:** Differentially methylated CpGs for each comparison were identified using the nested method in the Limma R package ^40^.

**Title: Supplementary Data 3 List of all significant meQTL associations for cluster-associated CpGs (FDR< 0.05)**.
**Description:** Statistical significance was evaluated using an additive linear model implemented in the matrix eQTL R package ^91^.
